# Improving mental well-being in psychocardiology—a feasibility trial for a non-blended web application as a brief metacognitive-based intervention in cardiovascular disease patients

**DOI:** 10.3389/fpsyt.2023.1138475

**Published:** 2023-09-28

**Authors:** Katharina Larionov, Ekaterina Petrova, Nurefsan Demirbuga, Oliver Werth, Michael H. Breitner, Philippa Gebhardt, Flora Caldarone, David Duncker, Mechthild Westhoff-Bleck, Anja Sensenhauser, Nadine Maxrath, Michael Marschollek, Kai G. Kahl, Ivo Heitland

**Affiliations:** ^1^Department of Psychiatry, Social Psychiatry and Psychotherapy, Hannover Medical School, Hannover, Germany; ^2^Information Systems Institute, Leibniz University Hannover, Hannover, Germany; ^3^OFFIS - Institute for Information Technology, Oldenburg, Germany; ^4^Department of Cardiology and Angiology, Hannover Medical School, Hannover, Germany; ^5^University of Applied Sciences and Arts, Hochschule Hannover, Hannover, Germany; ^6^TU Braunschweig and Hannover Medical School, Peter L. Reichertz Institute for Medical Informatics, Hannover, Germany

**Keywords:** psychocardiology, cardiovascular disease, anxiety, depression, mental health, metacognitive therapy, e-health, digital intervention

## Abstract

**Background:**

Many patients with cardiovascular disease also show a high comorbidity of mental disorders, especially such as anxiety and depression. This is, in turn, associated with a decrease in the quality of life. Psychocardiological treatment options are currently limited. Hence, there is a need for novel and accessible psychological help. Recently, we demonstrated that a brief face-to-face metacognitive therapy (MCT) based intervention is promising in treating anxiety and depression. Here, we aim to translate the face-to-face approach into digital application and explore the feasibility of this approach.

**Methods:**

We translated a validated brief psychocardiological intervention into a novel non-blended web app. The data of 18 patients suffering from various cardiac conditions but without diagnosed mental illness were analyzed after using the web app over a two-week period in a feasibility trial. The aim was whether a non-blended web app based MCT approach is feasible in the group of cardiovascular patients with cardiovascular disease.

**Results:**

Overall, patients were able to use the web app and rated it as satisfactory and beneficial. In addition, there was first indication that using the app improved the cardiac patients’ subjectively perceived health and reduced their anxiety. Therefore, the approach seems feasible for a future randomized controlled trial.

**Conclusion:**

Applying a metacognitive-based brief intervention via a non-blended web app seems to show good acceptance and feasibility in a small target group of patients with CVD. Future studies should further develop, improve and validate digital psychotherapy approaches, especially in patient groups with a lack of access to standard psychotherapeutic care.

## Introduction

Cardiovascular diseases (CVDs) belong to the largest group of civilization diseases worldwide and are the leading cause of mortality, representing 32% of all global deaths in 2019 (1). The associated life-threatening cardiac events, such as myocardial infarction, often lead to longstanding impacts upon people’s lives (2). The accompanied stress reactions frequently result in pathological anxiety and depressive symptoms, often to the point of psychiatric conditions (3). Among patients entering cardiac rehabilitation, 18% suffer from clinically significant depression and 28% report anxiety (4). Quite commonly, cardiac patients are absent from employment for long periods of time due to physical, but also psychological impairment (5, 6). This leads to a high level of subjective suffering and places a burden on the healthcare system (7). Furthermore, depression and anxiety disorders worsen adherence of cardiac patients during treatment and thus contribute to increased mortality (8). After all, CVDs and mental disorders amplify each other reciprocally, creating a vicious cycle (9). The initial psychological symptoms may worsen and manifest over time if not treated (10). As a result, longer and more intensive interventions and medications are necessary (10). In the present study, we focus on anxiety and depression as the two major psychological comorbidities in CVD patients.

Therefore, current cardiological guidelines pay special attention to the identification of psychological symptoms among CVD patients (8, 11). Given the need to explore treatment options for psychological conditions in CVD patients, a third-wave psychotherapy method has recently gained attention, metacognitive therapy (MCT) (12). Similar to CBT, MCT focusses on negative beliefs as a major contributor of psychopathology. However, MCT solely focuses on beliefs about one’s own cognition called metacognitions that determine the interpreting, monitoring, and controlling of the thinking process (12). The core of MCT is the Cognitive Attentional Syndrome (CAS), which is characterized by a perseverative type of thinking and inflexibility of attention. According to MCT, psychiatric symptoms occur when a person uses the CAS to process an ordinary stressful situation. Dysfunctional metacognitions will become entrenched, leading to even greater anxiety, worry and depressive thoughts (13). Those affected are caught in a self-reinforcing cycle and are mentally focused on their worries (14). The inflexibility of their attention control prevents them from breaking out of their symptoms. The effect of MCT on CVD patients has been investigated and demonstrated in several studies (15). Given that anxiety and rumination are common in cardiology patients, MCT appears to be successful in this group of patients due to the impact on dysfunctional metacognitions and coping strategies that support the CAS (16). MCT seeks to disrupt these supportive mechanisms by identifying their components and discussing their uselessness, as well as through several MCT techniques (12). One of them is called Attention Training Technique (ATT) and assumes that patients with psychiatric symptoms, such as anxiety and depression, cannot easily shift their attention, so they are unconsciously reinforcing pathological mechanisms (17). ATT is the first technique that helps break down the dysfunctional processes that support CAS. Another prominent method is Rumination/Worry Postponement, which challenges beliefs about the uncontrollability of worry and rumination (12). Through these and other techniques, patients are trained in detached Mindfulness (DM). In DM, patients have an observer role toward their own thoughts, they stop identifying with those thoughts and refrain from acting in response to them.

As of yet, there is evidence from several meta-analyses and RCTs in favor of face-to-face MCT (18, 19). However, for many patients, in-person visits can be a barrier. In addition, in Western countries, and especially in Germany, there are gaps in regional access to the therapy as well as lack of psychotherapists for the ever-increasing number of those in need (20–22). The SARS-CoV-2 pandemic has only worsened the deficit in psychological care (23, 24).

In this context, some studies have provided promising data, showing, that brief psychological interventions can help patients with CVDs get back into everyday activity (25). Recently, MCT has been also shown to be an encouraging approach for brief internet interventions (26, 27). In a recent case series from our own lab, patients with CVD and psychological distress underwent eight adapted video call sessions with a psychotherapist of 50 min each. These were designed to teach strategies for dealing with worry and rumination according to metacognitive therapy. The results of this study suggest that patients may benefit in terms of symptom reductions and an increase in metacognitive competence in themselves following these eight online video sessions (27).

Although brief MCT is effective and permits saving and rational use of available psychological aid resources, it is still face-to-face therapy and thus dependent on the availability of psychotherapists. The possibility of using digital solutions has already been explored for CBT (28–31). Comparisons have also been made as to whether a non-blended intervention is as effective as a blended one (32). The use of guided MCT for self-help in CVD was investigated by Wells et al. (15) in the recent PATHWAY program. In addition to video, audio, and text materials, patients were contacted by telephone by specially trained staff to discuss the content of the therapy. In evaluating the program, however, half of the patients indicated that they did not need the calls and even rated them negatively (26).

To our knowledge, metacognitive therapy using a digital intervention without psychological support has not been investigated. In this study, we developed a metacognitive therapy-based, non-blended web app specifically targeting patients suffering from CVD. The research objective of our study is to assess whether the approach of a web app is feasible.

## Methods

### Design

The present study was designed as an exploratory prospective observational single-arm feasibility trial. We here aim to translate a brief face-to-face metacognitive psychotherapy for CVD patients into the digital realm and explore the feasibility of that approach.

### Ethical approval

All study procedures were reviewed and approved by the local ethics committee of Hannover Medical School. Written informed consent in accordance with the Declaration of Helsinki was provided by all subjects prior to participation.

### Recruitment

**Figure 1:**
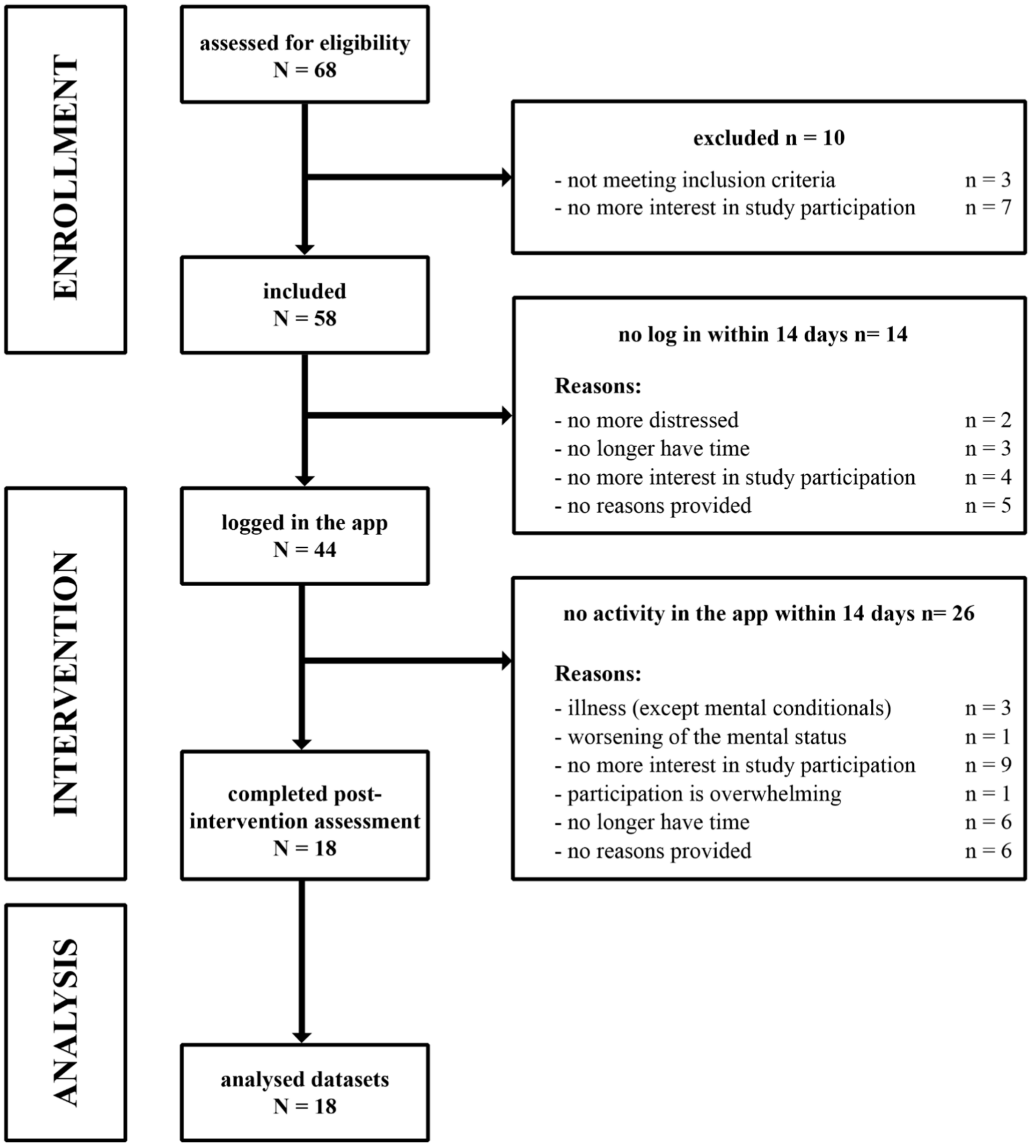
Patient flow during the study.

Participants currently suffering from a diagnosed CVD were recruited at the Department of Cardiology and Angiology of Hannover Medical School. Patients were informed by the cardiologists of the listed departments about the purpose of the study and were screened for the following inclusion criteria: (a) confirmed diagnosis of a CVD, (b) age 18–65 years, (c) no diagnosed mental illness (previously or currently existing) and no corresponding psychotherapeutic intervention during the study, (d) not having psychiatric medication or being stable at medication for at least 3 months, (e) the ability to use a digital device (i.e., smartphone, tablet or desktop-PC with an internet browser), (f) consistent access to the internet, (g) suitable knowledge of German language. The exclusion criteria were: (a) acute suicidal tendencies, (b) acute psychotic symptoms. Besides the baseline inclusion visit, the entire study was conducted remotely. Patient flow is shown in CONSORT flow chart (Figure 1). From the total of 68 recruited CVD patients, 58 met the inclusion criteria and were included in the study. Patients that did not log in within 14 days (*n* = 14) got excluded and did not participate in the study at all, meaning after signing the initial informed consent, no data could be collected. This does not only include data from our non-blended web app itself, but also all questionnaires both pre and post web app usage. The same applies to patients who had not edited any content 14 days after logging in (*n* = 26). After patients dropped out of the study, they were asked to provide feedback via email about their reasons for withdrawing.

### Study procedures

All participants received verbal and written instructions regarding study participation and app usage at the baseline visit. An access link to the web app and a personal key for the digital module-specific survey as well as brief instructions on the study procedure were provided via mail. The participants were asked to complete the baseline questionnaires before starting the training program. After the training, the questionnaires had to be filled out and returned by mail. Supportive phone calls by the study team were made once a week to inquire about the status of data processing and any possible technical problems related to the usage of the web app. In addition, technical email support was provided. There was no contact with a psychotherapist at any point. No financial compensation was provided for participation in the study.

### MCT in form of a non-blended web app

The web app is based on preliminary work by our research group, in which the German version of metacognitive therapy (MCT) manual (12) was used to create a brief intervention for patients with CVDs (27). The web app can be used by patients across different platforms and devices, e.g., a smartphone, tablet, or computer. It consists of two main parts, learning and practicing. Following the brief instructions in the email, a patient enters the learning section, which consists of six modules based on the described short-term MCT intervention (27). Each module includes five to eight psychoeducational videos in which an animated character guides participants through the training program. The first module is an introduction to MCT, where patients learn the specific terms used in the program, as well as the storylines and thought patterns of simulated patients who report on certain CVDs. The second module introduces cognitive attentional syndrome (CAS), a chain of mechanisms that support anxiety and rumination (33). This module is essential since, even in conventional therapy, identifying the CAS is a challenging task on which the clinical effect of MCT relies. The third module provides information on ATT. The video with instructions for the German version of the ATT in this Module recommends that patients, after watching it, perform the attention training daily, in parallel with the learning part. In the fourth module the basics of the CAS model are explained, using one cardiac patient’s CAS as an example. The fifth module covers techniques of DM. In addition, a new tool for addressing dysfunctional thoughts is presented—the Rumination/Worry Postponement and consolidates all learned materials and is followed by a glossary. Online ratings of the web app modules are collected using an external survey platform LimeSurvey (version 5.4.2; LimeSurvey GmbH., Hamburg, Germany) (34).

The practicing part of the app is structured threefold: (a) ATT, (b) DM, and (c) Rumination Postponement (33). If, when and how often subjects use the different exercises is a free choice of the user. Of note, the option to revisit materials is not restricted. During initial piloting of the app, it was shown that completion of all learning modules plus continued exercising as instructed takes approximately 14 days. The subjects were instructed to complete the learning part of the app in 14 days, but the access to the learning and practicing content of the app was not limited in time. The web app is only available in German language.

## Measures

### Hospital anxiety and depression scale

The Hospital Anxiety and Depression Scale (HADS) (35) is a self-reported tool for measuring the level of anxiety and depression in the past 7 days. It consists of 14 items, with seven each assigned to two subgroups. Each item is measured using a 4-point Likert scale from 0 to 3. A total maximum of 21 points is possible in each subscale. The summed points of the subscales correlate directly with the severity of anxiety and depression. HADS has good psychometric properties and is widely used on patients with somatic diseases (36, 37), including cardiology patients (38, 39). In our case, we used the German version (40).

### World Health Organization quality of life assessment

The World Health Organization Quality of Life Assessment (WHOQOL-BREF) (41) is a self-administered short version of the WHOQOL-100 (42) questionnaire and assesses an individual’s quality of life in the past 2 weeks based on four domains: physical health, psychological domain, social relationships, and environment. In addition, there are two questions assessing the overall quality of life and perceived general health. The questionnaire includes 26 questions on a 5-point Likert scale, ranging from complete disagreement (1) to complete agreement (5) with the statement. The sum scores of the domains are transformed into a scale of 0–100. Higher scores on the questionnaire correspond to a higher quality of life. The questionnaire has good content validity, internal consistency, and test–retest validity (41). The German version of the questionnaire was used (43).

### Metacognitions Questionnaire-30

We used the German translation of the Metacognitions Questionnaire-30 (MCQ-30; 32) is a self-administered measurement tool that asseses several metacognitive parameters divided into five domains, each consisting of six items: (1) positive beliefs about worry, (2) negative beliefs about worry concerning uncontrollability and danger, (3) low cognitive confidence, (4) need to control thoughts, (5) cognitive self-consciousness. It consists of 30 items containing Likert scale ratings (1 do not agree to 4 agree very much), with higher scores indicating more dysfunctional metacognitive beliefs. All subscales of the MCQ-30 were highly consistent in cardiology patients, as well as the total score (Cronbach’s alpha = 0.91) (44). The German version of the questionnaire was used (12).

### User version of the mobile application rating scale

The user version of the mobile application rating scale (uMARS) is the end-user version of the MARS (45) and represent a self-reported questionnaire to assess the quality of mobile health (mHealth) apps. It includes 20 items corresponding to five subscales: four for the objective quality: (a) engagement, (b) functionality, (c) esthetics, and (d) information quality and one subscale for the (e) subjective quality assessment. There is a further 6-items subscale for assessing the (f) user’s perceived impact of the evaluated app. Each item uses a 5-point scale as in original MARS (1-inadequate, 2-poor, 3-acceptable, 4-good, 5-excellent) (45). The item of the (e) subjective quality subscale may be answered as not applicable (N/A) and are, in that case, not added to the total score. The uMARS scoring is based on mean scores for each subscale. The internal consistency and test–retest reliability of the uMARS total score and subscales were shown as excellent in the RCT (Cronbach’s alpha = 0.90) (46). Here we used the German version of the uMARS, which uses items from the validated German version of the MARS (45)—MARS-G (47) includes similar items to the uMARS, so we adapted it for our study, using experience of other research groups (48, 49).

### Module specific survey

In addition to the uMARS, patients were asked to rate the content of each module on a 10-point scale directly after the respective module was completed. This questionnaire was integrated into the web app as a link to LimeSurvey (34). The questions can be found in Supplementary Figure S1.

### Statistical analysis

Exploratory analyses were conducted in SPSS software (version 29; IBM Corp., Armonk, NY, United States). uMARS and rating scale data are presented descriptively. As part of exploratory analyses within the context of our feasibility study, we investigated changes in WHOQOL-BREF, HADS and MCQ-30 from baseline to post web app using *t*-tests for dependent measures (alpha of 0.05, two-tailed). Note, that these analyses are intended to explore the feasibility of translating into a non-blended web app rather than rejecting or accepting a hypothesis as in a RCT.

## Results

### Sample description

Eighteen participants with documented CVD completed the trial. Demographic data and details on the underlying cardiac conditions are shown in Table 1.

**Table 1:** Participants’ demographic characteristics.

Demographic factors	Entire sample (*N* = 18)	*n* (%)/SD
**Gender**		
Male	10	56%
Female	8	44%
**Age, mean (SD)**	52	(11)
**Employment status**		
Employed	10	56%
Unemployed, retired	7	39%
Prefer not to say or N/A^a^	1	5%
**Educational qualification**		
Diploma/degree	2	11%
School/vocational	14	78%
Prefer not to say or N/A	2	11%
**Documented CVD** ^b^		
GUCH (grown-up congenital heart disease)	5	27%
Cardiac arrhythmia	3	17%
Heart failure with reduced ejection fraction	7	39%
Other (heart transplant, hypertrophic cardiomyopathy, idiopathic PAH)	3	17%
**Self-reported psychiatric disease**		
Yes	2	11%
No	16	89%
**Psychological therapy**		
In the past or planed	3	17%
Never	15	83%

a Not applicable.

b Cardiovascular disease.

### Web-app usage data

Participants took an average of 12 days (SD 13.6) to work through the learning area as shown in Table 2. The completion rate of the learning content was on average 91.2% (SD 18.8). Thirteen participants used the practicing area and completed it with the mean rate of 70.9% (SD 45.5).

**Table 2:** Average web app modules completion rates.

Item	Mean	(SD)	*n*^a^
Learning	91.2%	(18.8)	18
Practicing	70.9%	(45.5)	13
Module 1	95.7%	(9.4)	18
Module 2	98.8%	(3.6)	18
Module 3	89.1%	(7.6)	16
Module 4	86.5%	(7.1)	17
Module 5	88.9%	(7.6)	16
Module 6	80.6%	(9.2)	15
Glossary	66.7%	(11.4)	14
Web app usage duration (days)	12.2	(13.6)	18

a Entire sample (*N* = 18).

100% could be achieved by completing all five learning modules and watching the closing video in module six. The duration of web app usage counts the time from starting the first video in module one to finishing the last learning video in module five.

### Changes after web app-usage

**Figure 2:**
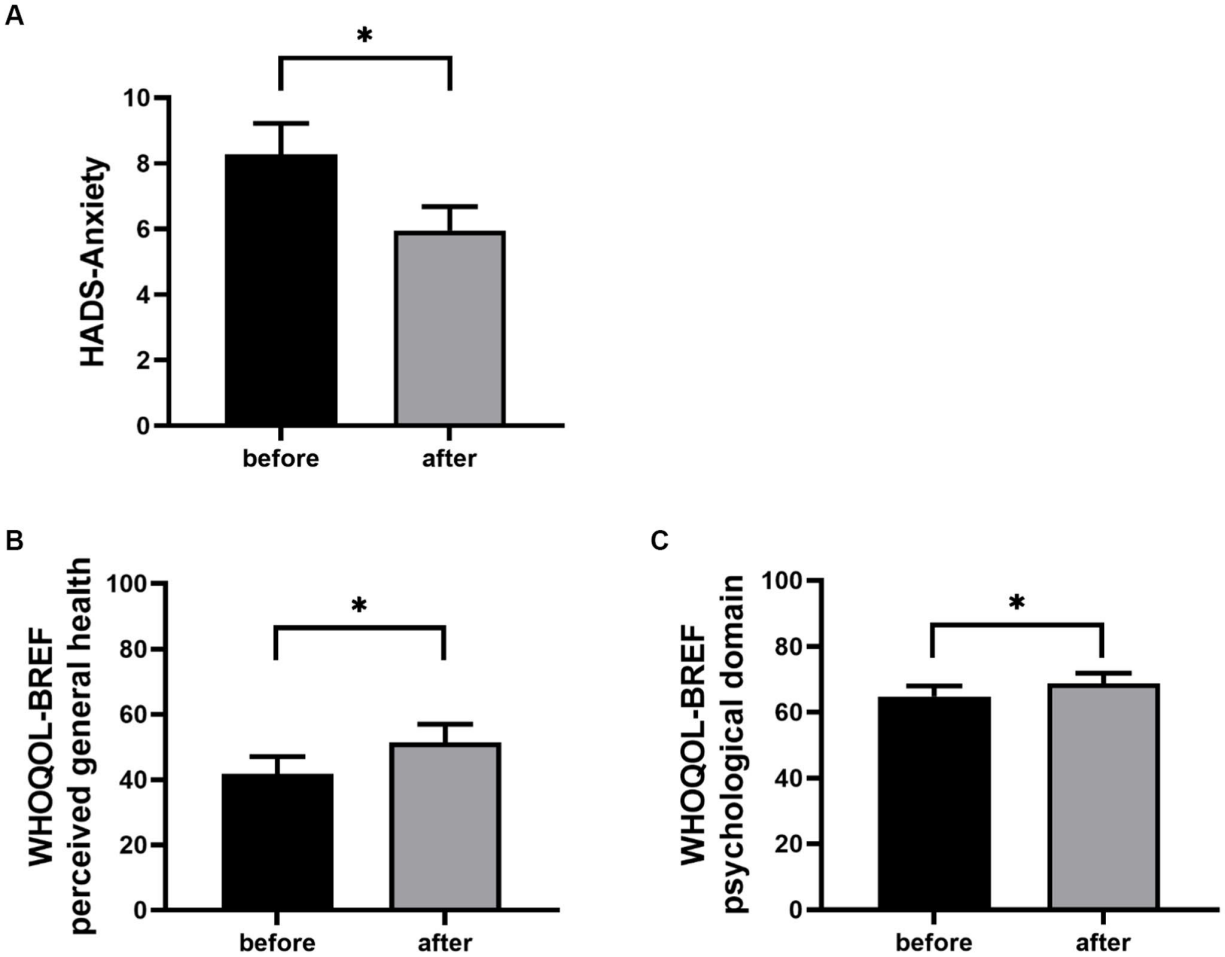
HADS-Anxiety **(A)**, WHOQOL-BREF perceived general health **(B)** and WHOQOL-BREF psychological domain **(C)** mean scores before and after using the web app are shown ±1 SEM. *p < 0.05.

After completing the web app modules, there was an improvement in the patients’ perceived general health [WHOQOL-BREF general health; *t* (17 = 2.72, *p* = 0.015, Cohen’s d = 0.64)] and in the psychological domain [WHOQOL-BREF psychological; *t* (16 = 2.23, *p* = 0.041, Cohen’s d = 0.54) see Figure 2]. Furthermore, anxiety decreases significantly from pre- to post-training [HADS-A; *t* (17 = 2.49, *p* = 0.02, Cohen’s d = 0.59)], while depression did not decrease [HADS-D; *t* (17 = 0.73, *p* = 0.48)]. Regarding changes in metacognitions, there was no change in the MCQ-30 total score [*t* (17 = 1.33, *p* = 0.20)]. The score in the MCQ-30 subscale cognitive self-consciousness changed from 12.78 to 11.78 [MCQ-30 cognitive self-consciousness; *t* (17 = 1.91, *p* = 0.07, Cohen’s d = 0.45)]. There were no effects regarding the other subscales of the MCQ-30 (all *p*-values > 0.21).

### Rating of the web app

**Figure 3:**
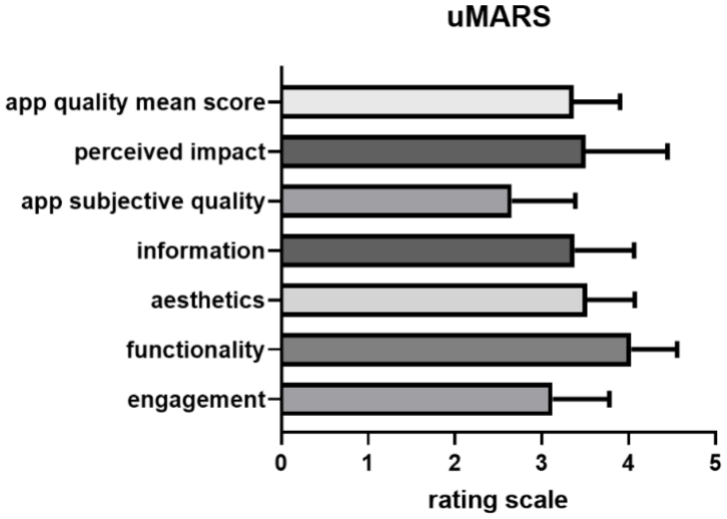
Participants uMARS ratings of the web app for all sections from 1 to 5 points are shown ±1 SEM.

The assessment of the general acceptability of the users regarding the web-app-based training program was based on the uMARS scale and the module-specific questions. An overview of the ratings of the perceived engagement, functionality, esthetics, information, app subjective quality, the user’s perceived impact and the app quality mean score of the evaluated app is presented in Figure 3. According to the uMARS scoring participants rated the app as acceptable on average (mean score of 3.1 from 5 possible points) (Table 3). The results of the module specific questionnaires are shown in Supplementary Figure S1. The results of uMARS participant’s individual feedback can be found in Supplementary Table S1.

**Table 3:** Assessment scores for baseline and post-intervention measures.

	Baseline		Post-intervention	
Scores	Mean	SD	Mean	SD
**World Health Organization Quality of Life Assessment (WHOQOL-BREF)**				
**Domains**				
Physical	59.73	18.40	62.5	18.81
Psychological*	64.71	13.43	68.7	12.86
Social relationships	60.65	19.76	62.5	21.25
Environment	75.20	14.79	76.6	10.76
Perceived QOL^a^	61.11	19.60	62.5	12.86
Perceived general health*	41.67	22.69	51.4	23.44
**Hospital Anxiety and Depression Scale (HADS)**				
**Subscales**				
HADS - Anxiety*	8.28	3.98	5.94	3.10
HADS - Depression	5.72	3.68	5.22	3.83
HADS - Total score	14.00	6.55	11.17	6.41
**Metacognitions Questionnaire-30 (MCQ-30)**				
**Subscales**				
Positive beliefs about worry	9.72	2.76	10.06	2.13
Negative beliefs about uncontrollability and danger of worry	11.22	4.14	10.83	3.82
Cognitive confidence	11.28	4.35	10.89	4.17
Need for control	12.22	4.41	11.06	3.37
Cognitive self-consciousness	12.78	3.95	11.78	3.06
MCQ-30 total score	57.22	16.09	54.61	13.09

a Quality of Life.

*p < 0.05.

## Discussion

Here, we developed an MCT-based brief intervention as a web app with learning content based on results of the former developed and tested face-to-face trial (27). We translated the individually tailored modular learning content into digital form, which includes video recording, creating animations and recording the audio content. Then, 18 patients diagnosed with a CVD tested the web app and provided extensive feedback on its functionality, the subjective quality of it and parts that may be improved. Moreover, anxiety and depression symptoms and metacognitive competency were assessed at baseline and after.

Overall, the web app approach seems to be accepted by users, with functionality rated best. On average, the participants needed 12 days to complete the training, however, with a range of 13.6 days. Among all the participants who engaged with the app, a moderate to high completion rate in the learning area was shown, while participants were more reluctant to practice. Additionally, we observed an improvement in the patient’s perceived health and psychological health, reduction in anxiety symptoms, as well as nonsignificant trend toward improvement in cognitive self-efficacy.

These initial results suggest that it quite might be feasible to deliver a MCT-based brief intervention to patients in the form of an app. However, the effects on efficacy need to be further explored in larger randomized controlled trials with a correspondingly larger number of participants. The personal feedback in the uMARS questionnaire from the complete datasets showed that the content of a web app could be processed by the participating CVD patients. The individual approach from the former trial seems to be well suited for this purpose (15). However, once again, further studies are needed in the future to appropriately examine the effect on larger and more heterogeneous groups. It would be interesting to investigate whether the initial nonsignificant trends shown in pre to post measures from the feasibility study can also be observed in larger numbers of cases. Especially given the shortage of psychotherapists, such non-blended web app-based approaches are promising to provide scalable opportunities in the future.

This is the first prototype of the web app, which still lacks, above all, the possibility of personalized customization. A plan for further development includes programming the web app in a way that allows each user to customize a learning path for themselves.

### Effects on depression and anxiety

Patients with CVD often suffer from pronounced anxiety, whether it is fear of reinfarction in patients after myocardial infarction, or otherwise (50). A digital tool capable of reducing anxiety and easily accessible at any time, especially when experiencing acute symptoms of anxiety, offers a solid backup option beyond the clinical setting. This could be an important strength of future digital apps, making them complementary to professional psychological treatment.

In our study, we were able to observe first effects with regard to symptom improvement. At baseline, CVD patients showed mild anxiety symptoms. After the use of the web app, anxiety symptoms were much less pronounced on average. This initially promising data should be replicated and validated, preferably using a larger patient sample as well as a randomized and controlled design.

Before the start of the intervention, no depression symptoms were measurable in the participants. After app usage no decrease could be proven here either. Hence, a floor effect seems likely with regard to no effect of the web app on depression. Future studies in CVD patients with and without depression are needed to fully evaluate potential effects of such web apps on it.

For the MCQ-30 scale a non-significant trend in cognitive self-awareness could be observed. However, more data is needed using similar web app designs, preferably compared to traditional CBT or MCT interventions to shed light on the working mechanisms of the improvements in symptomology. Currently, the modules of the web app have a uniform design for all users. In previous studies, authors suggested that a specific MCT intervention should be adapted for any type of CVD separately to be able to address the pathomechanisms of mental disorders more individually (12, 27). The lack of significant changes in participants’ metacognitive status can also be due to further reasons. It may mean that the learning content was not understood, completion rates in exercises were too low, or that participants had no previous pathological metacognitions. Integrating better and clearer instructions into the web app is one possible solution. Since at least improvement tendencies have been shown, it is possible that the effects of MCT techniques are too low in healthy individuals. Therefore, a larger study involving patients with a CVD and anxiety and depression assessed with validated psychological instruments may provide additional insights.

### User behavior

The major aim during development was to bring a face-to-face intervention into a digital learning program, with a structure that allows the training to be completed entirely non-blended and to communicate the challenging content of the MCT-based learning modules to patients in the most comprehensible way.

Overall patients made substantial progress in the training program, and we registered a mean completion rate of 91.2% in the learning area calculated from the rates of all 18 participants and a mean completion rate of 70.9% performed by at least 13 participants. The remaining five participants did only complete the modular learning area. Wells et al. (26) showed a completion rate of 50–70% of at least four modules while being guided. This supports the assumption that CVD patients are quite capable of handling digital learning. We observed that the patients comprehended the structure of the web app prototype and worked through the learning content completely on their own. There was no additional guidance from the study team at any point. For this reason, the accepted app structure should be retained as far as possible and slightly adapted. Areas for learning and for practice should continue to be separated, and the modular structure should be retained. Likewise, the basic elements, such as introduction, learning section, and summary and repetition of learning content should remain consistent in each module.

However, the effect of MCT results from the confluence of learning and practice together, suggesting that practice must be adequate and regular. Dammen et al. (51) demonstrated that ATT exercise, which is also a part of our web app in particular leads to significant improvement in anxiety and depression symptoms. The exercise area was introduced in a module in the middle of the training program where the participants were instructed to practice regularly. From that point onwards, participants had access to the practice area at any time and could practice at their own discretion. Unfortunately, our data on details on for the lack of motivation to practice is limited. We received some hints from the uMARS optional comment section, which can be found in Supplementary Table S1. While three patients reported being satisfied with the exercises, two participants lacked details about the mechanisms of action of the exercises. One participant described the exercises as too extensive. Furthermore, three patients missed interactive parts, as well as gamification elements. Another patient would like a tool to record progress after exercises.

These points must be considered in the development of future web apps. Overall, the importance of practicing must be made especially clear to users. A revision of the explanatory video, as well as implemented reminders upon completion of each learning module, could remedy this. Patients could also receive daily motivating messages on their mobile devices tracking their progress and encouraging them to continue practicing. Furthermore, an integrated concept of gamification could increase the overall motivation of the patients. At present, interactive elements are rarely used in existing mental health but patients seem to wish for it according to feedback (52). In addition, the link to the exercises in the learning area should be integrated more frequently, as some patients described that they were confused and did not know when exactly to practice.

In the present study, we also experienced an increase in anxiety and depression in two male patients. The first patient left the study after completing the learning part at 100%. The reason was that using the app and intensely interacting with the own negative thoughts caused him to feel poorer. The second patient left the study for the same reasons as the first but returned to the study 4 weeks later and completed it. Both patients were contacted by a psychotherapist and were provided with support and recommendations. Thus, future research should consider risk factors for deterioration in order to prevent them. At this point, it is not irrelevant that studies devoted to psychological aid publish positive results, while deterioration of psychological well-being remains unreported (53). As with any other effective intervention, psychotherapy contains the potential for deterioration of the mental state of the patient (54), which is necessarily discussed before the start of the intervention. Regarding the negative effects of Internet-based therapy in anxiety and depression among patients in the treatment group, adverse effects can vary from 4 to 10% (55, 56). It is important to note that in the control group, side effects are reported more frequently, suggesting that the benefits of therapy outweigh the possible negative effects (57, 58).

For the sake of completeness, it should be noted that we noticed two particularly critical points within the patient flow, as shown in Figure 1. Out of a total sample of 58, 14 patients did not log into the web app and of those who did log in, 22 did not start the training. The reasons for this varied widely. The most frequently cited reasons were no longer having interest in study participation (*n* = 13) and no longer having time (*n* = 9), while 11 patients did not want to give a reason. Just from these results, one must ask about the motivation of the patients to participate in such a training and how it could be increased. Since the participants were not proven to have depression or anxiety, the important next step is to investigate the motivation for training in a patient group with a higher level of psychological distress.

### Technical implementation

The technical implementation of the first version was rated overall with three out of five points, which shows that there is still room for improvement here in any case. The functionality of the prototype was rated the best suggesting that the web app works satisfyingly, the menu is clearly structured and thus comprehensible navigation is provided.

On this basis, it is possible to improve the attractiveness of other web app focuses, that have received a lower rating, like the visual quality of the web app, which was rated with 3.5 of 5 points. A uniform design must be created professionally here, which must be a necessary process in the development of a native app in any case. Multimedia content, i.e., graphics, animations, videos and sound recordings, must either be revised or completely recreated based on the original content.

### Limitations

Some general aspects of study design should be considered and changed in future studies. A larger sample size and comparison to a randomized control group could provide further information to generalize the results. However, the purpose of this study was primarily to evaluate the functionality of the web app and if its application is feasible. In future, these preliminary results can be used as a basis for appropriate changes to provide patients with the most efficient tool possible.

The second point is the limitations of the web app. Depending on the web platform provider, it is possible to get a wide range of functionality sufficient to create a training program. However, the web app provides limited opportunities to create interactive elements, such as gamification (59). Many internet interventions use interactive elements to maintain motivation and reinforce the desired effect of the intervention (60). In addition, regular reminders that appear directly on the end device could increase activity and participation (61). As the present study had multiple dropouts, it is necessary to develop a native mobile app to overcome the disadvantages of the web app described above.

Another point is the diversity of the sample. The study was focused on patients with various CVD pathology, and the presence of depression and anxiety was not an obligatory criterion for inclusion. Thus, the effects of the app-based MCT in the sample with anxiety and depression need to be investigated. Further research should also consider gender matching since women are more inclined to ruminate than men (62). Furthermore, different age groups participated in our study (mean age 52, SD 11). Research indicates that CVD patients <56 years old were four times more likely to use any mobile technology than those >69 years old and three times more likely to use technology for medical purposes (63). Regarding the technology use levels, some participants generally use more mobile devices and apps than others. However, some potential participants had neither mail addresses nor internet access. These factors should be taken into account in the planning of subsequent studies.

## Conclusion

The feasibility study suggests that a non-blended metacognitive therapy-based web app may offer a promising approach for future randomized controlled trials. The results of the study can be improved by developing a native app, with more options for customization to meet the needs of CVD patients. In addition, future research should focus on further exploring the app and its effects based on the results of this study in larger trials.

## Data availability statement

The original contributions presented in the study are included in the article/Supplementary material, further inquiries can be directed to the corresponding author.

## Ethics statement

The studies involving humans were approved by ethics board of Hanover Medical School, Hanover. The studies were conducted in accordance with the local legislation and institutional requirements. The participants provided their written informed consent to participate in this study.

## Author contributions

KL and EP were responsible for study design and technical implementation, conducted the study, performed data collection and processing, statistical analysis, and drafted the manuscript. DD and MW-B implemented and supervised the data collection. The informatics implementation was carried out by OW, ND, NM, AS, MB, and MM. KK was responsible for study design, conduct of the study, and data interpretation. IH was responsible for study design, conduct of the study, statistical analysis, data interpretation, and draft of the manuscript. All authors contributed to the article and approved the submitted version.

## Funding

This study was supported by Else Kröner-Fresenius-Stiftung (Promotionsprogramm DigiStrucMed 2020_EKPK.20).

## Conflict of interest

The authors declare that the research was conducted in the absence of any commercial or financial relationships that could be construed as a potential conflict of interest.

## Publisher’s note

All claims expressed in this article are solely those of the authors and do not necessarily represent those of their affiliated organizations, or those of the publisher, the editors and the reviewers. Any product that may be evaluated in this article, or claim that may be made by its manufacturer, is not guaranteed or endorsed by the publisher.

## References

[1] World Health Organization (WHO). The WHO Global NCD action plan 2013–2020. (2013). Available at: https://www.who.int/publications/i/item/9789241506236 (Accessed December 6, 2022).

[2] Kirchberger I Heier M Amann U Kuch B Thilo C Meisinger C. Variables associated with disability in male and female long-term survivors from acute myocardial infarction. Results from the MONICA/KORA myocardial infarction registry. Prev Med. (2016) 88:13–9. doi: 10.1016/j.ypmed.2016.03.009, PMID: 27002251

[3] Hare DL Toukhsati SR Johansson P Jaarsma T. Depression and cardiovascular disease: a clinical review. Eur Heart J. (2013) 35:1365–72. doi: 10.1093/eurheartj/eht462, PMID: 24282187

[4] Rao A Zecchin R Newton PJ Phillips JL DiGiacomo M Denniss AR. The prevalence and impact of depression and anxiety in cardiac rehabilitation: a longitudinal cohort study. Eur J Prev Cardiol. (2020) 27:478–89. doi: 10.1177/2047487319871716, PMID: 31597473

[5] Johnston DA Harvey SB Glozier N Calvo RA Christensen H Deady M. The relationship between depression symptoms, absenteeism and presenteeism. J Affect Disord. (2019) 256:536–40. doi: 10.1016/j.jad.2019.06.041 31280078

[6] Söderman E Lisspers J Sundin Ö. Depression as a predictor of return to work in patients with coronary artery disease. Soc Sci Med. (2003) 56:193–202. doi: 10.1016/S0277-9536(02)00024-2, PMID: 12435561

[7] Timmis A Vardas P Townsend N Torbica A Katus H de Smedt D. European Society of Cardiology: cardiovascular disease statistics 2021. Eur Heart J. (2022) 43:716–99. doi: 10.1093/eurheartj/ehab892, PMID: 35016208

[8] Visseren FLJ Mach F Smulders YM Carballo D Koskinas KC Bäck M. 2021 ESC guidelines on cardiovascular disease prevention in clinical practice. Eur Heart J. (2021) 42:3227–337. doi: 10.1093/eurheartj/ehab484 34458905

[9] Albus C Waller C Fritzsche K Gunold H Haass M Hamann B. Significance of psychosocial factors in cardiology: update 2018: position paper of the German cardiac society. Clin Res Cardiol. (2019) 108:1175–96. doi: 10.1007/s00392-019-01488-w, PMID: 31076853

[10] Bukh JD Bock C Vinberg M Kessing LV. The effect of prolonged duration of untreated depression on antidepressant treatment outcome. J Affect Disord. (2013) 145:42–8. doi: 10.1016/j.jad.2012.07.008, PMID: 22854096

[11] Richards SH. Psychological interventions for coronary heart disease. Cochrane Database Syst Rev. (2017) 4:Cd002902. doi: 10.1002/14651858.CD002902.pub2 28452408 PMC6478177

[12] Wells A. Metakognitive Therapie bei Angststörungen und Depression, vol. 1. Basel: Beltz (2011).

[13] Korn O Rudolf S. Sorgenlos und grübelfrei: Wie der Ausstieg aus der Grübelfalle gelingt. In: Selbsthilfe und Therapiebegleitung mit Metakognitiver Therapie. Basel: Beltz Verlag (2015).

[14] Wells A. Breaking the cybernetic code: understanding and treating the human metacognitive control system to enhance mental health. Front Psychol. (2019) 10:2621. doi: 10.3389/fpsyg.2019.02621 31920769 PMC6920120

[15] Wells A Reeves D Heal C Davies LM Shields GE Heagerty A. Evaluating metacognitive therapy to improve treatment of anxiety and depression in cardiovascular disease: the NIHR funded PATHWAY research Programme. Front Psychol. (2022) 13:886407. doi: 10.3389/fpsyt.2022.886407, PMID: 35722590 PMC9204153

[16] McPhillips R Salmon P Wells A Fisher P. Qualitative analysis of emotional distress in cardiac patients from the perspectives of cognitive behavioral and metacognitive theories: why might cognitive behavioral therapy have limited benefit, and might metacognitive therapy be more effective? Front Psychol. (2018) 9:2288. doi: 10.3389/fpsyg.2018.02288 30662413 PMC6328488

[17] Wells A. Panic disorder in association with relaxation induced anxiety: an attentional training approach to treatment. Behav Ther. (1990) 21:273–80. doi: 10.1016/S0005-7894(05)80330-2

[18] Normann N Morina N. The efficacy of metacognitive therapy: a systematic review and Meta-analysis. Front Psychol. (2018) 9:2211. doi: 10.3389/fpsyg.2018.02211, PMID: 30487770 PMC6246690

[19] Callesen P Reeves D Heal C Wells A. Metacognitive therapy versus cognitive behaviour therapy in adults with major depression: a parallel single-blind randomised trial. Sci Rep. (2020) 10:7878. doi: 10.1038/s41598-020-64577-1, PMID: 32398710 PMC7217821

[20] BPtK-Auswertung: Monatelange Wartezeiten bei Psychotherapeut*innen. Available at: https://www.bptk.de/bptk-auswertung-monatelange-wartezeiten-bei-psychotherapeutinnen/#:~:text=Nach%20einer%20BPtK%2DAuswertung%20von,sind%20und%20deshalb%20behandelt%20werden (Accessed December 8, 2022).

[21] Singer S Maier L Paserat A Lang K Wirp B Kobes J. Wartezeiten auf einen Psychotherapieplatz vor und nach der Psychotherapiestrukturreform. Psychotherapeut. (2022) 67:176–84. doi: 10.1007/s00278-021-00551-0

[22] Bleckmann W.M.-B. Haluka. Wartezeiten für Psychotherapieplätze sind weit höher als von Krankenkassen angegeben. (2022). Available at: https://www.rbb24.de/panorama/beitrag/2022/05/wartezeiten-psychotherapie-laenger-als-angaben-krankenkassen.html (Accessed Dezember 10, 2022).

[23] World Health Organization. COVID-19 pandemic triggers 25% increase in prevalence of anxiety and depression worldwide. Available at: https://www.who.int/news/item/02-03-2022-covid-19-pandemic-triggers-25-increase-in-prevalence-of-anxiety-and-depression-worldwide (Accessed August 12, 2022). PMC9998057 35414629

[24] American Psychological Association. Psychologists struggle to meet demand amid mental health crisis. (2022). Available at: https://www.apa.org/pubs/reports/practitioner/2022-covid-psychologist-workload (Accessed Dezember 9, 2022).

[25] Winter L Naumann F Olsson K Fuge J Hoeper MM Kahl KG. Metacognitive therapy for adjustment disorder in a patient with newly diagnosed pulmonary arterial hypertension: a case report. Front Psychol. (2020) 11:143. doi: 10.3389/fpsyg.2020.00143, PMID: 32116944 PMC7028769

[26] Wells A Reeves D Heal C Fisher P Doherty P Davies L. Metacognitive therapy self-help for anxiety-depression: single-blind randomized feasibility trial in cardiovascular disease. Health Psychol. (2022) 41:366–77. doi: 10.1037/hea0001168, PMID: 35467904 PMC9037049

[27] Gebhardt P Caldarone F Westhoff-Bleck M Olsson KM Hoeper MM Park DH. Metacognitive short-term intervention in patients with mental disorders following cardiovascular events. Front Psych. (2022) 13:812807. doi: 10.3389/fpsyt.2022.812807, PMID: 35444582 PMC9013742

[28] Svärdman F Sjöwall D Lindsäter E. Internet-delivered cognitive behavioral interventions to reduce elevated stress: a systematic review and meta-analysis. Internet Interv. (2022) 29:100553. doi: 10.1016/j.invent.2022.100553, PMID: 35781929 PMC9240371

[29] Lundgren JG Dahlström Ö Andersson G Jaarsma T Kärner Köhler A Johansson P. The effect of guided web-based cognitive behavioral therapy on patients with depressive symptoms and heart failure: a pilot randomized controlled trial. J Med Internet Res. (2016) 18:e194. doi: 10.2196/jmir.5556, PMID: 27489077 PMC5070581

[30] Păsărelu CR Andersson G Bergman Nordgren L Dobrean A. Internet-delivered transdiagnostic and tailored cognitive behavioral therapy for anxiety and depression: a systematic review and meta-analysis of randomized controlled trials. Cogn Behav Ther. (2017) 46:1–28. doi: 10.1080/16506073.2016.1231219, PMID: 27712544

[31] Norlund F Wallin E Olsson EMG Wallert J Burell G von Essen L. Internet-based cognitive behavioral therapy for symptoms of depression and anxiety among patients with a recent myocardial infarction: the U-CARE heart randomized controlled trial. J Med Internet Res. (2018) 20:e88. doi: 10.2196/jmir.9710, PMID: 29519777 PMC5874001

[32] Krämer R Köhne-Volland L Schumacher A Köhler S. Efficacy of a web-based intervention for depressive disorders: three-arm randomized controlled trial comparing guided and unguided self-help with waitlist control. JMIR Form Res. (2022) 6:e34330. doi: 10.2196/34330, PMID: 35105536 PMC9016501

[33] Faßbinder E Klein JP Sipos V Schweiger U. Therapie-Tools Depression. Weinheim Basel: Beltz-Verlag (2015):167–69.

[34] LimeSurvey. Available at: https://www.limesurvey.org/de/ (Accessed November 27, 2022).

[35] Zigmond AS Snaith RP. The hospital anxiety and depression scale. Acta Psychiatr Scand. (1983) 67:361–70. doi: 10.1111/j.1600-0447.1983.tb09716.x, PMID: 6880820

[36] Herrmann C. International experiences with the hospital anxiety and depression scale-a review of validation data and clinical results. J Psychosom Res. (1997) 42:17–41. doi: 10.1016/S0022-3999(96)00216-4, PMID: 9055211

[37] Bjelland I Dahl AA Haug TT Neckelmann D. The validity of the hospital anxiety and depression scale. An updated literature review. J Psychosom Res. (2002) 52:69–77. doi: 10.1016/S0022-3999(01)00296-3 11832252

[38] Haddad M Walters P Phillips R Tsakok J Williams P Mann A. Detecting depression in patients with coronary heart disease: a diagnostic evaluation of the PHQ-9 and HADS-D in primary care, findings from the UPBEAT-UK study. PLoS One. (2013) 8:e78493. doi: 10.1371/journal.pone.0078493, PMID: 24130903 PMC3795055

[39] Martin CR Lewin RJ Thompson DR. A confirmatory factor analysis of the hospital anxiety and depression scale in coronary care patients following acute myocardial infarction. Psychiatry Res. (2003) 120:85–94. doi: 10.1016/S0165-1781(03)00162-8, PMID: 14500117

[40] Hermann-Lingen C Buss U Snaith RP. Hospital anxiety and depression scale - deutsche version. Deutsche adaptation der Hospital anxiety and depression scale (HADS) von R. P. Snaith und a. S. Zigmond. Bern: Hans Huber (2011).

[41] The WHOQOL Group. Development of the World Health Organization WHOQOL-BREF quality of life assessment. The WHOQOL Group Psychol Med. (1998) 28:551–8. doi: 10.1017/S0033291798006667 9626712

[42] The WHOQOL Group. The World Health Organization quality of life assessment (WHOQOL): development and general psychometric properties. Soc Sci Med. (1998) 46:1569–85. doi: 10.1016/S0277-9536(98)00009-4 9672396

[43] Angermeyer RK Matschinger H. WHOQOL-100 und WHOQOL-BREF. Handbuch für die deutschsprachigen Versionen der WHO Instrumente zur Erfassung von Lebensqualität. (2000).

[44] Faija CL Reeves D Heal C Wells A. Metacognition in cardiac patients with anxiety and depression: psychometric performance of the metacognitions questionnaire 30 (MCQ-30). Front Psychol. (2020) 11:1064. doi: 10.3389/fpsyg.2020.01064, PMID: 32528387 PMC7264260

[45] Stoyanov SR Hides L Kavanagh DJ Zelenko O Tjondronegoro D Mani M. Mobile app rating scale: a new tool for assessing the quality of health Mobile apps. JMIR Mhealth Uhealth. (2015) 3:e27. doi: 10.2196/mhealth.3422, PMID: 25760773 PMC4376132

[46] Stoyanov SR Hides L Kavanagh DJ Wilson H. Development and validation of the user version of the Mobile application rating scale (uMARS). JMIR Mhealth Uhealth. (2016) 4:e72. doi: 10.2196/mhealth.5849, PMID: 27287964 PMC4920963

[47] Messner E-M Terhorst Y Barke A Baumeister H Stoyanov S Hides L. The German version of the Mobile app rating scale (MARS-G): development and validation study. JMIR Mhealth Uhealth. (2020) 8:e14479. doi: 10.2196/14479, PMID: 32217504 PMC7148545

[48] Lambrecht A Vuillerme N Raab C Simon D Messner EM Hagen M. Quality of a supporting Mobile app for rheumatic patients: patient-based assessment using the user version of the Mobile application scale (uMARS). Front Med (Lausanne). (2021) 8:715345. doi: 10.3389/fmed.2021.715345, PMID: 34368202 PMC8339429

[49] Lull C von Ahnen JA Gross G Olsavszky V Knitza J Leipe J. German Mobile apps for patients with psoriasis: systematic search and evaluation. JMIR Mhealth Uhealth. (2022) 10:e34017. doi: 10.2196/34017, PMID: 35617014 PMC9185339

[50] Palacios J Khondoker M Mann A Tylee A Hotopf M. Depression and anxiety symptom trajectories in coronary heart disease: associations with measures of disability and impact on 3-year health care costs. J Psychosom Res. (2018) 104:1–8. doi: 10.1016/j.jpsychores.2017.10.015, PMID: 29275777

[51] Dammen T Tunheim K Munkhaugen J Papageorgiou C. The attention training technique reduces anxiety and depression in patients with coronary heart disease: a pilot feasibility study. Front Psychol. (2022) 13:948081. doi: 10.3389/fpsyg.2022.948081, PMID: 35967654 PMC9363691

[52] Brown M O'Neill N van Woerden H Eslambolchilar P Jones M John A. Gamification and adherence to web-based mental health interventions: a systematic review. JMIR Ment Health. (2016) 3:e39. doi: 10.2196/mental.5710, PMID: 27558893 PMC5014987

[53] Barlow DH. Negative effects from psychological treatments: a perspective. Am Psychol. (2010) 65:13–20. doi: 10.1037/a0015643 20063906

[54] Hansen NB Lambert MJ Forman EM. The psychotherapy dose-response effect and its implications for treatment delivery services. Clin Psychol Sci Pract. (2002) 9:329–43. doi: 10.1093/clipsy.9.3.329

[55] Cuijpers P Reijnders M Karyotaki E de Wit L Ebert DD. Negative effects of psychotherapies for adult depression: a meta-analysis of deterioration rates. J Affect Disord. (2018) 239:138–45. doi: 10.1016/j.jad.2018.05.050, PMID: 30005327

[56] Ebert DD Donkin L Andersson G Andrews G Berger T Carlbring P. Does internet-based guided-self-help for depression cause harm? An individual participant data meta-analysis on deterioration rates and its moderators in randomized controlled trials. Psychol Med. (2016) 46:2679–93. doi: 10.1017/S0033291716001562, PMID: 27649340 PMC5560500

[57] Rozental A Magnusson K Boettcher J Andersson G Carlbring P. For better or worse: an individual patient data meta-analysis of deterioration among participants receiving internet-based cognitive behavior therapy. J Consult Clin Psychol. (2017) 85:160–77. doi: 10.1037/ccp0000158 27775414

[58] Rozental A Andersson G Carlbring P. In the absence of effects: an individual patient data Meta-analysis of non-response and its predictors in internet-based cognitive behavior therapy. Front Psychol. (2019) 10:10. doi: 10.3389/fpsyg.2019.00589 30984061 PMC6450428

[59] Cheng VWS. Recommendations for implementing gamification for mental health and wellbeing. Front Psychol. (2020) 11:586379. doi: 10.3389/fpsyg.2020.586379, PMID: 33365001 PMC7750532

[60] Cheng VWS Davenport T Johnson D Vella K Hickie IB. Gamification in apps and Technologies for Improving Mental Health and Well-Being: systematic review. JMIR Ment Health. (2019) 6:e13717. doi: 10.2196/13717, PMID: 31244479 PMC6617915

[61] Nahum-Shani I Smith SN Spring BJ Collins LM Witkiewitz K Tewari A. Just-in-time adaptive interventions (JITAIs) in Mobile health: key components and design principles for ongoing health behavior support. Ann Behav Med. (2018) 52:446–62. doi: 10.1007/s12160-016-9830-8, PMID: 27663578 PMC5364076

[62] Johnson DP Whisman MA. Gender differences in rumination: a meta-analysis. Pers Individ Dif. (2013) 55:367–74. doi: 10.1016/j.paid.2013.03.019, PMID: 24089583 PMC3786159

[63] Gallagher R Roach K Sadler L Glinatsis H Belshaw J Kirkness A. Mobile technology use across age groups in patients eligible for cardiac rehabilitation: survey study. JMIR Mhealth Uhealth. (2017) 5:e161. doi: 10.2196/mhealth.8352, PMID: 29066425 PMC5676027

