# Supplementary material for: Improving mental well-being in psychocardiology—a feasibility trial for a non-blended web application as a brief metacognitive-based intervention in cardiovascular disease patients
Source: Front Psychiatry. 2023 Sep 28;14:1138475. doi: 10.3389/fpsyt.2023.1138475 (PMC10568139; doi:10.3389/fpsyt.2023.1138475)
Supplement: Supplementary file 1 [file Data_Sheet_1.PDF]

Supplementary Material

Improving mental well-being in psychocardiology – development and validation of the unguided web application based on metacognitive therapy in cardiovascular disease patients

Katharina Larionov*, Ekaterina Petrova*, Nurefsan Demirbuga, Oliver Werth, Michael H. Breitner, Philippa Gebhardt, Flora Caldarone, David Duncker, Mechthild Westhoff-Bleck, Anja Sensenhauser, Nadine Maxrath, Michael Marschollek, Kai G. Kahl*, Ivo Heitland*

Correspondence: Ivo Heitland: Heitland.ivo-aleksander@mh-hannover.de

Supplementary Figures and Tables

FIGURE 4. Selected relevant questions from the Module Specific Survey.

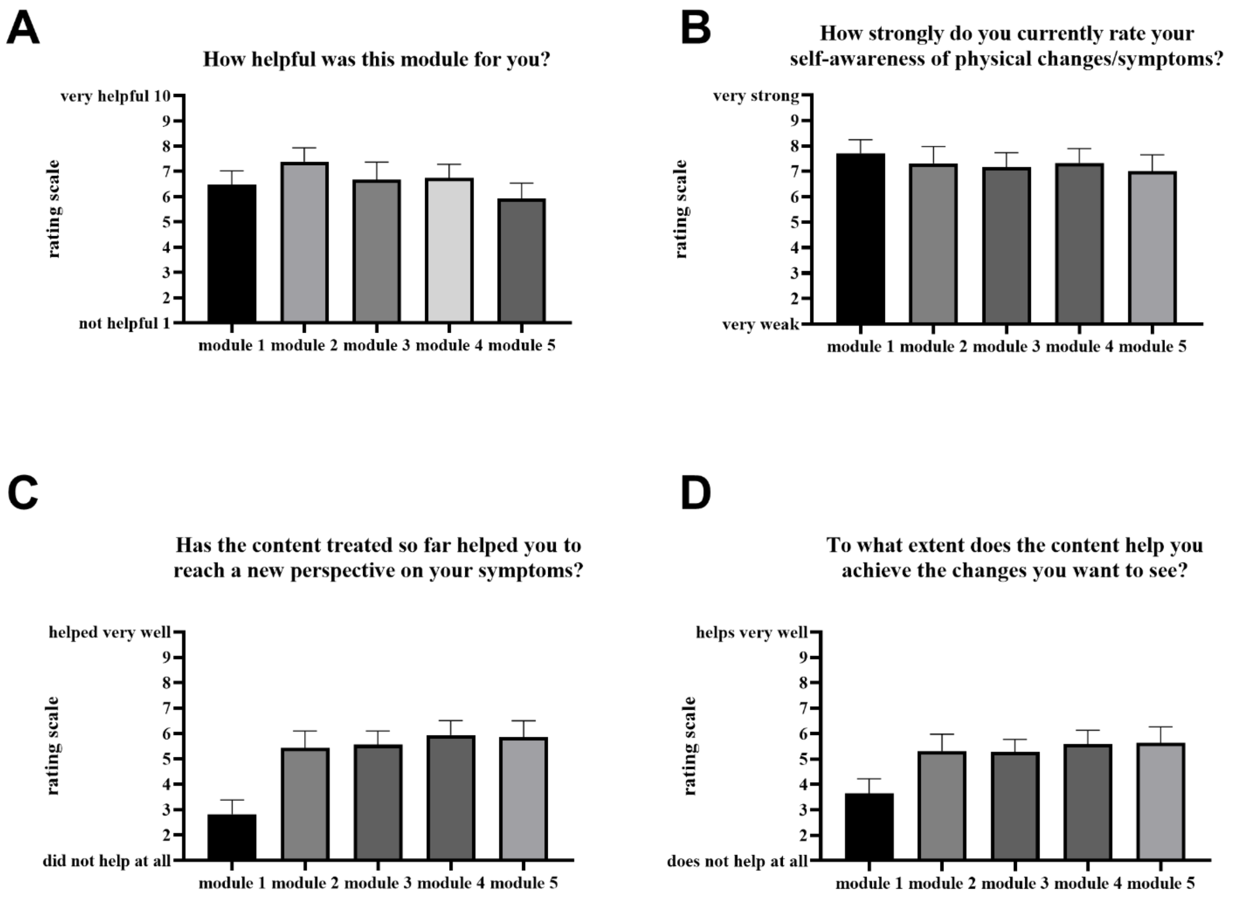

TABLE 4. uMARS participan’s text feedback divided into categories according to context of meaning.

Items	n	%
**Benefits of the web app**		
Idea of the app good	6	33%
Accessibility: quickly available when you need help	2	11%
Exercises are good, especially DM	3	17%
Provides new perspectives on the structure of thinking	1	6%
App is understandable and easy to use	2	11%
Sample persons and their problems are real, easy to reflect on them	3	17%
**Areas to be improve**		
Interactive parts missing – need for	3	17%
Moderate quality of the graphics, animation and sound	6	33%
Progress tracking tools after exercises missing	1	6%
Lack of details of working mechanisms of the exercises	2	11%
Exercises too extensive	1	6%
Some web app - platform related issues	2	11%
No feedback provided	4	22%

Entire sample (N = 18)
